# Six Shades of Vascular Smooth Muscle Cells Illuminated by KLF4 (Krüppel-Like Factor 4)

**DOI:** 10.1161/ATVBAHA.121.316600

**Published:** 2021-09-02

**Authors:** Carmen Yap, Arnout Mieremet, Carlie J.M. de Vries, Dimitra Micha, Vivian de Waard

**Affiliations:** Department of Medical Biochemistry, Amsterdam Cardiovascular Sciences, University of Amsterdam, Amsterdam UMC, Location Academic Medical Center, The Netherlands (C.Y., A.M., C.J.M.d.V., V.d.W.).; Department of Clinical Genetics, Amsterdam Cardiovascular Sciences, Vrije Universiteit Amsterdam, Amsterdam UMC, Location VU University Medical Center, Amsterdam, The Netherlands (D.M.).

**Keywords:** atherosclerosis, cardiovascular disease, myocytes, phenotype, smooth muscle

## Abstract

Multiple layers of vascular smooth muscle cells (vSMCs) are present in blood vessels forming the media of the vessel wall. vSMCs provide a vessel wall structure, enabling it to contract and relax, thus modulating blood flow. They also play a crucial role in the development of vascular diseases, such as atherosclerosis and aortic aneurysm formation. vSMCs display a remarkable high degree of plasticity. At present, the number of different vSMC phenotypes has only partially been characterized. By mapping vSMC phenotypes in detail and identifying triggers for phenotype switching, the relevance of the different phenotypes in vascular disease may be identified. Up until recently, vSMCs were classified as either contractile or dedifferentiated (ie, synthetic). However, single-cell RNA sequencing studies revealed such dedifferentiated arterial vSMCs to be highly diverse. Currently, no consensus exist about the number of vSMC phenotypes. Therefore, we reviewed the data from relevant single-cell RNA sequencing studies, and classified a total of 6 vSMC phenotypes. The central dedifferentiated vSMC type that we classified is the mesenchymal-like phenotype. Mesenchymal-like vSMCs subsequently seem to differentiate into fibroblast-like, macrophage-like, osteogenic-like, and adipocyte-like vSMCs, which contribute differentially to vascular disease. This phenotype switching between vSMCs requires the transcription factor KLF4 (Krüppel-like factor 4). Here, we performed an integrated analysis of the data about the recently identified vSMC phenotypes, their associated gene expression profiles, and previous vSMC knowledge to better understand the role of vSMC phenotype transitions in vascular pathology.

**Highlights**

- Single-cell RNA sequencing and lineage tracing analyses revealed that vascular smooth muscle cells can be identified beyond the synthetic and contractile profile.
- We discuss the phenotypic switching of vascular smooth muscle cells from a contractile state to a mesenchymal-like, fibroblast-like, macrophage-like, osteogenic-like, or adipocyte-like phenotype.
- The transcription factor KLF4 (Krüppel-like factor 4) plays a pivotal role in regulation of vascular smooth muscle cells phenotype switching and emerges as potential drug target.
- The current challenge is to pinpoint the diverging roles of vascular smooth muscle cell phenotypes and their impact on the broad range of cardiovascular diseases.

Cardiovascular diseases are the leading cause of death worldwide. Many of these pathologies are characterized by progressive cellular modulations derived from genetic predisposition, aging, or lifestyle.^1^ The 3 main cellular layers forming the vessel wall are the adventitia, media, and the intima, surrounding the lumen. In the media, the middle layer, vascular smooth muscle cells (vSMCs) are the major cellular component. These vSMCs contribute to the integrity of the vessels and are able to adequately respond to stimuli of vasoconstriction and vasodilation.^2,3^ In healthy vessels, vSMCs are regarded as quiescent, differentiated cells, which display remarkable plasticity.^4^ The contractile vSMCs are subject to context-dependent changes and studies have shown that a subset of vSMCs in healthy tissue express reduced levels of the contractile vSMC markers, capable of phenotypic switching.^4^ There is a growing body of evidence that multiple vSMC phenotypes exist in healthy vessels. Single-cell RNA sequencing (scRNA-seq) studies identified the presence of specific vSMC-derived cell populations.^5–9^ However, there is still limited understanding of the role of these vSMC phenotypes in physiological and pathological conditions.

For decades, vSMC activation and dedifferentiation has been regarded as the adoption of a single synthetic, proliferative phenotype. However, as revealed by recent scRNA-seq analyses, the diversity of vSMC phenotypes is far more sophisticated.^10^ The power of scRNA-seq is that it provides detailed, unbiased information on distinct cell populations within healthy or diseased tissue. Distinct cell populations were identified by scRNA-seq in the vessel wall, with the majority concerning immune cells, such as T cells, B cells, myeloid cells, and mast cells. Nonimmune cells in atherosclerotic plaques and aortic aneurysm tissue included endothelial cells and vSMCs. The combination of scRNA-seq and lineage tracing is extremely useful as it allows in-depth vSMC phenotypic characterization.^10^ With the development of lineage tracing in mouse models, the fate of vSMCs can be accurately tracked and provides detailed information of their phenotypic modulation and contribution to diseased tissue.^11,12^ In this review, we highlighted the information obtained about vSMCs and used the recent scRNA-seq literature to classify 6 different vSMC phenotypes. Besides a contractile phenotype, we distinguish the mesenchymal-like, fibroblast-like, macrophage-like, osteogenic-like, and adipocyte-like phenotypes. In addition, we address the current insights about stimulating and inhibitory cues mediating vSMC phenotype switching. From that analysis, we inferred that transcription factor KLF4 (Krüppel-like factor 4) plays a pivotal role in the initial dedifferentiation of vSMCs to the mesenchymal-like phenotype enabling further cellular changes toward the other 4 vSMC phenotypes.

## Contractile vSMCs

In a healthy state, vSMCs in the media of the arterial vessel wall actively synthesize, secrete, modulate, and maintain the extracellular matrix (ECM) to provide elasticity and strength to the blood vessel. In the vasculature, the vSMCs work in close collaboration with other cells to counterbalance the strong mechanical forces that the vessel wall experiences.^13,14^ A single layer of endothelial cells at the luminal side of the vessel forms the tight barrier between vascular lumen and the vessel wall, providing cues for vSMC relaxation and contraction. Endothelial cell function is determined by shear stress, stretching of the vessel due to heart pulse, and by circulating factors.^13^ In the adventitia, fibroblasts produce and maintain collagen fibers, thereby forming a peripheral structure to preserve vascular integrity at high pressures.^14^ In the adventitia of larger blood vessels, a microvasculature bed is present, termed the vasa vasorum. This structure protrudes into the outer layers of the media to provide sufficient oxygen and nutrients to the multi-layered vSMCs in the media.^13^

Contractile vSMCs are regarded as differentiated and quiescent cells under physiological conditions, expressing a panel of typical contractile proteins that is crucial to maintain vascular tension.^15^ Although the embryonic origin of vSMCs is diverse, the contractile vSMC phenotype is considered universal throughout the arterial vasculature.^16^ Contractile vSMCs are embedded in an intricate structure of ECM composed of elastin and collagens as key fibers.^13,14,17^ The ECM is produced by vSMCs themselves and sequesters growth factors, such as those belonging to the TGF-β (transforming growth factor beta) family.^18^ Upon damage of the vessel wall, the sequestered growth factors are released to induce a local repair response.^19^

Contractile vSMCs exhibit an elongated, spindle-shaped morphology and express a well-characterized set of contractile markers including smooth muscle actin (*ACTA2*), smooth muscle myosin heavy chain (*MYH11*), smooth muscle protein 22-alpha (*SM22α/TAGLN*), smoothelin (*SMTN*), and calponin (*CNN1*).^20–22^ Expression of these proteins is controlled by the transcription factors MYOCD (myocardin) and SRF (serum response factor), both of which are involved in the regulation of differentiation to contractile vSMCs.^23^ These transcription factors also induce expression of microRNA cluster (miRNA)-143/145, involved in activation of the vSMC contractile phenotype.^24^ In addition, external stimuli, such as TGF-β and heparin, play a pivotal role in promoting and maintaining the vSMCs contractile phenotype. Exposure to these factors leads to upregulation of structural ECM genes and inhibition of vSMC proliferation and migration.^23,25^ The phosphatase and tensin homolog (PTEN) also helps to maintain the contractile vSMC phenotype, as in the nucleus PTEN interacts with SRF to enhance SRF binding to essential promoter elements in vSMC contractile genes.^26^ MEF2C (myocyte enhancer factor 2C) was also shown to be essential for contractile vSMC differentiation.^27^ A detailed overview of the contractile vSMC phenotype in the vasculature is given elsewhere.^20,22,28^

Various scRNA-seq analyses confirmed that contractile vSMCs are the most prominent cell type in the healthy vessel wall with the transcriptomic profile just described.^5,6,8^ Yet, in combination with lineage tracing, additional vSMC populations have been identified with distinct gene expression profiles.^5,6,8^ Once pathological processes in the vessel wall are initiated, vSMCs respond by changing their phenotype and function. A plethora of pathological cues induce these changes: factors from the circulation, compounds and proteins produced by activated endothelial cells, fibroblasts, perivascular adipocytes or inflammatory cells, (lack of) mechanical stress, damaged ECM protein fragments, or ECM-derived growth factors. In the next sections, we combine the information on the different phenotypes of vSMCs as deduced from the scRNA-seq reports with the available knowledge on specific cues and signal transduction pathways involved in vSMC phenotype modulation.

## Mesenchymal-Like vSMCs

Extensive lineage tracing studies revealed that contractile vSMCs can switch from a contractile status to a mesenchymal-like phenotype.^29–31^ A mesenchymal-like vSMC is characterized by the ability to proliferate and self-renew and is marked by reduced expression of contractile proteins. This phenotype overlaps with that of mesenchymal stem cells, which are defined as stromal cells that have the capacity to differentiate into multiple lineages.^29,32^ Recently, a significant number of scRNA-seq studies confirmed that among the various vSMC phenotypes identified, mesenchymal-like vSMCs are also present (Table). The selected scRNA-seq studies were performed on human and murine vascular tissue of atherosclerotic plaques or aortic aneurysms. Distinct vSMC clusters were specified, and based on their gene expression profile and available knowledge of upstream modulators, we aimed to identify the main drivers of phenotypic switches. In this review, we classified stem cell marker positive vSMCs as belonging to the mesenchymal-like phenotype. This included the pioneer cell phenotype of Alencar et al^33^ and vSMC-derived intermediate cells (SEM) by Pan et al.^8^

**Table.**
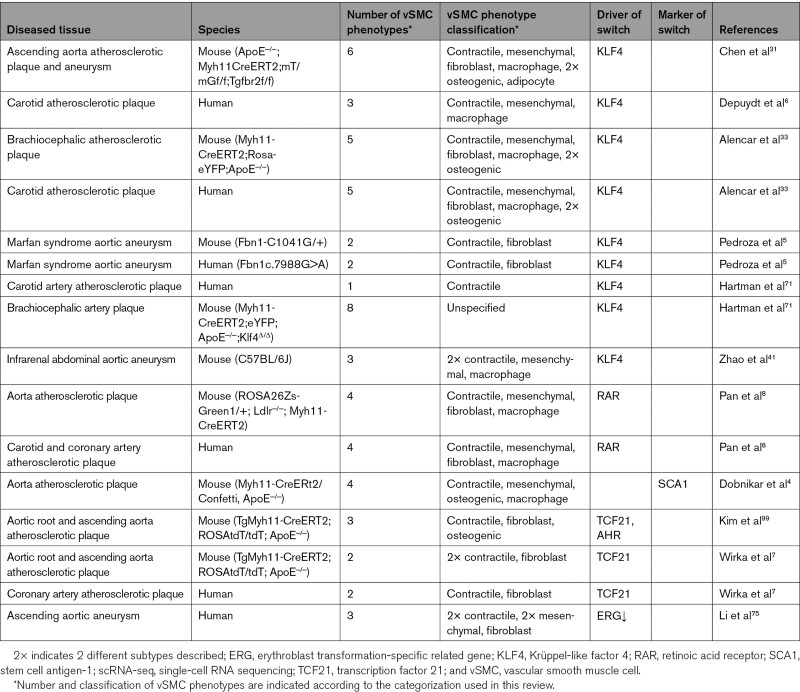
Overview of scRNA-Seq Studies That Identified vSMC Phenotypes

Dedifferentiation of vSMCs from a contractile to a mesenchymal-like phenotype is driven by external stimuli that induce repair and/or proliferation. A key initiating factor for this phenotypic switch is transcription factor KLF4,^29,34–38^ which regulates cellular proliferation and dedifferentiation. KLF4 also has a crucial function in the induction of cellular pluripotency.^39^ In fact, generation of induced pluripotent stem cells from a wide range of somatic cells requires KLF4 and 3 other, so-called Yamanaka, factors: OCT4 (octamer transcription factor 4), SOX2 (SRY-BOX transcription factor 2) and cMYC (MYC proto-oncogene, bHLH transcription factor).^39^

Induction of KLF4 in vSMCs results in a phenotypic switch from contractile to mesenchymal-like and initiates the expression of mesenchymal markers such as stem cell antigen-1 (SCA1)/*LY6A*, CD34, and CD44.^40,41^ During this transition, while gaining expression of mesenchymal markers, the contractile vSMCs lose expression of their contractile markers.^12,36,42^ Under physiological conditions, a subpopulation of mesenchymal-like vSMCs resides in the medial and adventitial layer of the arterial wall.^36,43,44^ Upon injury, mesenchymal-like vSMCs proliferate and migrate into the media and intima, to support tissue repair, possibly leading to neointimal thickening.^43,45–47^ Expression of the mesenchymal-like vSMC marker SCA1/*LY6A* increases in vSMCs cultured in vitro, after carotid artery ligation and in a subset of vSMCs in atherosclerotic plaques.^4^ Interestingly, a recent genetic fate mapping study reported only minimal involvement of SCA1/*LY6A*-positive vSMCs in atherosclerotic neointima formation, suggesting an injury- and/or context-dependent role of the SCA1/*LY6A* vSMC population.^48^ To further investigate the plasticity of human vSMCs switching to mesenchymal-like vSMCs as a source of reparative vSMCs, it is important to identify the human SCA1/*LY6A* orthologue. This will establish whether the mesenchymal-like vSMC population is also a prerequisite for tissue regeneration in human vascular disease.^49^

In several mouse vascular disease models, KLF4 signaling has been shown to induce vSMC differentiation toward a mesenchymal-like vSMC type in a context-dependent manner.^36,50–55^ In the adventitia, a population of vSMC-derived SCA1/*LY6A*-positive cells is formed upon induction of KLF4.^36^ Targeted deletion of KLF4 in vSMCs resulted in the elective loss of these vSMC-derived adventitial SCA1/*LY6A*-positive cells, but not of non-vSMC-derived adventitial SCA1/*LY6A*–positive cells. Consistent with these in vivo findings, KLF4 overexpression in cultured vSMCs promotes the formation of a progenitor cell phenotype with loss of vSMC differentiation markers.^36^ Moreover, KLF4 overexpression inhibits expression of vSMC contractile markers,^56^ possibly by repressing the expression of MYOCD.^53^

KLF4 is regulated by various signaling complexes at transcriptional and posttranslational levels.^57,58^ After exposure to PDGF-BB (platelet-derived growth factor BB), a stimulus of vSMC proliferation and phenotype switching, elevated levels of KLF4 were identified.^21,54,59,60^ PDGF-BB induces KLF4 expression via its receptor and subsequent transcription factor Sp1 (stimulating protein-1) activation.^55^ Interestingly, Sp1 also enhances *ACTA2* expression via TGF-β1 signaling.^61^ TGF-β1 is known to induce vSMC contractile proteins via activation of specific SMAD transcription factor family members,^61^ promoting the vSMC contractile phenotype.^62^ Indeed, deficiency of SMAD3 disrupts TGF-β signaling and decreases gene expression of the contractile vSMC phenotype markers.^63^ Thus, there seems to be a dual role for Sp1 in vSMC phenotype modulation. PDGF-BB also induced DOCK2 (dedicator of cytokinesis 2) and Olfm2 (olfactomedin 2).^64,65^ DOCK2 inhibits MYOCD-induced vSMC marker promoter activity. In addition, DOCK2 and KLF4 cooperatively inhibit MYOCD-SRF interaction.^64^ Olfm2 promotes the interaction of SRF with RUNX2 (runt-related transcription factor 2), leading eventually to reduction of vSMC marker gene transcription and consequently vSMC phenotypic modulation.^65^

KLF4 activity is modulated by retinoic acid as well.^8,50,66^ Retinoic acid and PDGF-BB have opposite effects on vSMC proliferation and differentiation by changing the phosphorylation and acetylation state of KLF4 in different ways, causing it to preferentially bind to different regions within the TAGLN promoter.^50,66–68^ Retinoic acid receptor activation leads to the phosphorylation of KLF4, thereby facilitates its acetylation and subsequent translocation to different transcriptional activation domains, alleviating the repression of contractile gene expression.^50^

Furthermore, it has been reported in human vSMCs, human tissue, and mouse models that the DNA-modifying enzyme TET2 (ten-eleven translocation-2) is upregulated in contractile vSMCs and reduced in dedifferentiation vSMCs. Knockdown of TET2 inhibited expression of MYOCD and SRF with transcriptional upregulation of KLF4, thus preventing vSMC differentiation to contractile vSMCs.^69^ Another member of the Krüppel family, namely ZFP148 (zinc finger protein 148) also modulates vSMC phenotype transition by inhibiting vSMC contractile marker expression in a neurofibromin 1-dependent manner.^27,70^

In line with the observation that vSMC phenotype switching is context-dependent, sex differences have also been detected in a recent scRNA-seq study comparing atherosclerotic plaque tissue of males and females.^71^ Different gene expression profiles were observed with a more pronounced KLF4-driven pattern in females compared with males. In a vSMC-specific KLF4 knockout mouse model, expression of several female-biased genes was observed (*FN1* was downregulated; *MFAP4*, *CNN1*, *NDRG2*, *GAS6*, and *OSR1* were upregulated).^71^ Such sex-specific differences in vSMC phenotype are undervalued and require more attention in future studies.

The role of miRNAs in the (de)differentiation of vSMCs has been highlighted in dedicated reviews.^51,52^ MiR-143 and miR-145 are crucial for the fate of vSMCs, facilitating vSMC differentiation and inhibiting proliferation.^51,52^ MiRNA-143/145 are controlled by transcription factors NKx2-5 (NK2 transcription factor related locus 5) and SRF with its coactivator MYOCD and target KLF4, KLF5, and Sp1.^52^ Deficiency of miRNA143/145 in mice resulted in thinning of the aortic wall and a reduced number of contractile vSMCs, indicating that miR-143/145 are important in maintaining the contractile vSMC phenotype.^51,72^ In addition, miR-1 repressed KLF4 to regulate vSMC differentiation. Specifically, miR1 expression was increased during differentiation of pluripotent embryonic stem cells toward vSMCs, and inhibition of miR1 repressed vSMC differentiation.^51^

To prevent or reverse the phenotypic transition to mesenchymal-like vSMCs, the expression of KLF4 can be suppressed by TGF-β or miR-143/145 to maintain the contractile vSMC phenotype.^24,73,74^ Indeed, treatment of mesenchymal-like vSMCs with TGF-β for 3 days increased ACTA2 expression, indicating an adoption of a more contractile phenotype.^8^ Also, the transcription factor erythroblast transformation-specific related gene, which is involved in handling reactive oxygen species and endoplasmic reticulum (ER) signaling, was decreased in mesenchymal-like vSMCs.^75^ Increasing the expression of erythroblast transformation-specific related gene, which plays an important role in maintaining normal aortic wall function, promotes the contractile vSMC phenotype.^75^ Alternatively, mesenchymal-like vSMC may undergo further changes into other vSMC phenotypes.^31^

## Fibroblast-Like vSMCs

Fibroblasts are cells, which are primarily responsible for the production and modification of ECM proteins, such as collagens and fibronectin throughout tissues.^76,77^ In response to injury, fibroblasts can transition into myofibroblasts, which generate collagen-rich scar tissue to repair a wound. Subsequently, extensive remodeling will take place to resolve the scar.^76,78–80^ Most scRNA-seq studies have reported fibroblasts-like vSMCs as a phenotype (Table). Generally, the fibroblast-like vSMC phenotype is observed following vascular injury, such as during atherosclerotic progression or aortic aneurysm formation. This phenotype is also referred to as myofibroblasts-like vSMCs or fibromyocytes.^7,81^

Fibroblast-like vSMCs shift toward, but cluster separately from, authentic fibroblasts in scRNA-seq analyses performed in atherosclerotic lesions of murine and human arteries.^7^ Fibroblast-like vSMCs express *ACTA2*, SCA1/*LY6A*, and the fibroblast markers lumican (*LUM*), biglycan (*BGN*), and decorin (*DCN*). The cells show substantially reduced levels of contractile markers (*TAGLN*, *CNN1*) as compared with contractile vSMCs.^7,82^ Phenotype switching to a fibroblast-like vSMC was observed in the thoracic aortic aneurysm of a Marfan syndrome mouse model.^5^ Profiling of gene expression in that cluster revealed enhanced expression of genes involved in adhesion, ECM organization, cellular proliferation, and deposition of collagen. The latter is considered a hallmark for aortic aneurysm formation, which involves aortic fibrosis and stiffness.

A number of factors have been identified that induce this fibroblast-like vSMC phenotype, summarized below. It has been shown that adventitial SCA1/*LY6A*–positive fibroblast-like vSMCs arise in a KLF4-dependent manner. The adventitial SCA1/*LY6A*-derived fibroblast-like vSMCs may even migrate into the intima where they promote a fibrotic response, which stiffens the vessel wall.^83^ Interestingly, cholesterol or oxidized phospholipid exposure can also induce a phenotypic switch of vSMCs as shown by induced expression of fibroblast and macrophage markers.^40^ Fibroblast markers upregulated under these conditions were fibronectin 1 (*FN1*), Ecrg4 augurin precursor (*ECRG4*), proteoglycan 4 (*PRG4*), secreted phosphoprotein 1 (*SPP1*), lipocalin-2 (*LP2*), metalloproteinase inhibitor 1 (*TIMP1*), *BGN*, and *DCN*.^40^

Intriguingly, the ER unfolded protein response (UPR) can promote phenotypic switching in vSMCs toward the fibroblast-like phenotype as well.^40^ The ER normally has a low cholesterol content, while accumulation of free cholesterol in the ER induces membrane dysfunction and subsequent ER stress, causing UPR.^84,85^ Moreover, the UPR promotes a macrophage-like vSMC phenotype, which may explain the appearance of both fibroblast-like and macrophage-like vSMCs in atherosclerotic plaques.^40^ Even without cholesterol exposure, chemically induced UPR is sufficient to cause phenotype switching of vSMCs to fibroblast-like or macrophage-like vSMCs.^40^ The underlying mechanism of this phenotypic switch is linked to the UPR effector ATF4 (activating transcription factor 4). ATF4 prevents proteasomal degradation of KLF4, and this enhanced KLF4 expression promotes atherosclerotic plaque formation.^40^

The vSMC-specific knockout of TCF21 (transcription factor 21) in hyperlipidemic apolipoprotein E deficient (ApoE^−/−^) mice led to fewer fibroblast-like vSMCs in the protective fibrous cap of the atherosclerotic lesions.^7^ Moreover, high TCF21 expression is associated with decreased coronary artery disease risk in humans, possibly due to a more stable and fibroblast-like vSMC-rich plaque. In addition, TCF21 is activated early on in coronary artery disease and directly inhibits SMAD3-mediated gene expression, thereby reducing expression of the contractile markers.^86^ Together, these studies point to TCF21 as a regulator of vSMC phenotype switching toward a protective fibroblast-like vSMC population in atherosclerosis.

The vSMC phenotypes distinct from the contractile type are often described as modulated vSMCs.^7,8^ Gene expression profiles of these modulated vSMCs comprise markers of mesenchymal-like and fibroblast-like but also of macrophage-like or osteogenic-like cells.^7,8,40^ Many fibroblast-like vSMCs originate from the mesenchymal-like pool of vSMCs, although it is challenging to distinguish between these phenotypes.^87^ It is also not clear if all fibroblast-like vSMCs first, and entirely, transition via the mesenchymal-like vSMC state. Therefore, it cannot be concluded that the fibroblast-like vSMCs are part of mesenchymal-like vSMCs or an entirely separate cell type without additional research. However, as the gene expression patterns still differ, currently, a distinction between the 2 cell states has been made.

Reverse differentiation from a fibroblast-like phenotype back to the contractile vSMCs phenotype has been described for DKK3 (dickkopf 3), which is a Wingless-related integration site (Wnt) inhibitor. DKK3 was shown to be involved in the differentiation of stem and progenitor cells to contractile vSMCs in ApoE^−/−^ mice.^88^ DKK3 induces differentiation of SCA1/*LY6A*-positive mesenchymal-like and fibroblasts-like vSMCs to contractile vSMCs via activation of TGF-β in a Wnt-dependent manner. While TCF21 seems to reduce atherosclerosis severity by promoting the fibroblast-like vSMC, DKK3 promotes protective atherosclerotic plaque stabilization by increasing the number of contractile vSMCs. Therefore, it is not yet clear which vSMC phenotype is actually beneficial in the context of atherosclerosis.

## Macrophage-Like vSMCs

Macrophages play a central role in all stages of inflammation and healing and recruit other immune cells to initiate an appropriate immune response to clear debris and combat pathogens.^89^ In atherosclerosis, macrophages are known to clear the vascular wall of oxidized LDL (low-density lipoprotein), in the process becoming foam cells.^90^ As stated earlier, vSMCs can acquire a macrophage-like phenotype, even with phagocytic properties,^91^ as reported in 6 out of eleven selected scRNA-seq studies (Table). Phenotype switching from a contractile vSMC, via the mesenchymal-like to a macrophage-like vSMC, is associated with development of atherosclerosis.^29,91,92^

Macrophage-like vSMCs are typically indicated by expression of *LGALS3* and classical macrophage markers such as CD11b, CD45, CD68, CD116, and F4/80 (for murine macrophages).^93^ Phenotype switching to macrophage-like vSMCs also involves KLF4 signaling.^29,91,92^ A conditional KLF4 knockout in an atherosclerotic mouse model results in reduced vSMC-derived mesenchymal-like cells and macrophage-like cells, plus a marked reduction in lesion size and increased plaque stability.^29,33^ Similar macrophage-like vSMCs have been identified in human atherosclerotic plaques.^6,8,33,71^ A low number of cells within the vSMC group was KLF4 positive, indicating that vSMCs probably only have transient KLF4 expression. One of these vSMC clusters was characterized by *ACTA2*, *LGALS3*, and *CD68* expression, typical for the macrophage-like phenotype.^6^ However, identification of macrophage-like cells is often based on different macrophage or foam cell markers between the scRNA-seq studies, with *LGALS3* and *CD68* being the most common markers.

KLF4 gene expression in vSMCs facilitates foam cell formation by enhancing the uptake of cholesterol-rich lipoproteins.^94^ While classically lipid-laden foam cells were considered to be solely derived from monocytes/macrophages, it has become evident that vSMCs with a macrophage-like phenotype are abundantly present in the atherosclerotic plaque.^94^ Upon high cholesterol exposure, expression of KLF4 was induced. This transforms contractile vSMCs into macrophage-like vSMCs, a cell population contributing to disease progression.^95^ Switching of vSMCs to a macrophage-like phenotype coincides with loss of the contractile vSMC factor MYOCD.^91,96,97^ Conversely, gain of MYOCD expression inhibits macrophage-like vSMC accumulation in atherosclerotic lesions in vivo.^91^ One of the Yamanaka factors, OCT4, also plays a role in regulating vSMC phenotypic transition, but interestingly in a contrasting way compared with KLF4. Deficiency of KLF4 or OCT4 resulted in opposite patterns of gene expression in vSMCs. During atherosclerosis, deficiency of KLF4 showed reduced lesion size, while OCT4 deficiency showed an increase in lesion size. Decreased lesion size was in turn consistent with increased plaque stability.^33,98^

Various scRNA-seq analyses have been performed on atherosclerotic plaques of mice.^4,7,8,31,33,99^ In ApoE^−/−^ mice fed a western diet, vSMCs expressing *Lgals3* compose up to two thirds of all vSMCs in the atherosclerotic lesion.^33^ This macrophage-like phenotype was similarly found by others in ApoE^−/−^ mice,^29,31,33,90,93^ as well as in LDL receptor knockout (LDLR^−/−^) mice.^8^ Through fate mapping, it appears that these macrophage-like vSMCs were derived from a multipotent vSMC-derived intermediate cell state,^8^ which bear similarity to the mesenchymal-like vSMCs. Recent evidence even suggests that a considerable subset of the plaque may originate from dedifferentiated vSMCs, which proliferate in a clonal fashion.^100^

Other extracellular stimuli playing a role in phenotypic switching to macrophage-like vSMCs are nitric oxide and natriuretic peptides. In response to these compounds, vSMCs generate cyclic GMP, which induces vasodilation, enhancing blood flow.^101^ Interestingly, PRKG1 (cyclic GMP–dependent protein kinase 1) activation also contributes to the formation of macrophage-like vSMCs that reside within the atherosclerotic plaque.^101^ Under atherogenic conditions, vSMCs migrate to the atherosclerotic plaque (intima), which in *Prkg1*-deficient mice remain in the medial layer. Using cell-fate mapping, it was shown that *Prkg1* is involved in phenotype switching of vSMCs to macrophage-like vSMCs in the plaques. In line with this, postnatal ablation of *Prkg1* in murine vSMCs resulted in smaller lesions.^101^ This study also demonstrates that macrophage-like vSMCs are derived from mature vSMCs that migrated into the plaque.^101^

Reverse differentiation of the macrophage-like vSMC phenotype is accomplished by HDL (high-density lipoprotein). HDL is responsible for the efflux of cholesterol from cells and transport to the liver to remove cholesterol from the periphery. Exposure to HDL reduces the macrophage-like vSMC phenotype by increasing MYOCD and miR-143/145 expression in vSMCs in vitro.^95^ The question remains whether macrophage-like vSMCs have similar functions as monocyte-derived macrophage subsets in atherosclerosis.^8^

## Osteogenic-Like vSMCs

The natural function of chondrocytes is to form and maintain cartilage, while that of osteoblasts is to generate bone tissue.^102,103^ During ossification, hypertrophic chondrocytes produce a unique ECM that mineralizes, enabling cells to differentiate into osteoblasts. Both chondrocytes and osteoblasts are of mesenchymal origin and share a common precursor.^104^ Switching from a mesenchymal-like vSMC phenotype to a chondrocyte-like or osteoblast-like vSMC has been identified in 4 out of the 11 scRNA-seq studies (Table). In this review, we classified chondrocyte-like and osteoblast-like vSMC phenotypes together as osteogenic-like vSMCs.

Deposition of calcium phosphate can drive vSMC phenotype switching to contribute to various cardiovascular diseases.^105,106^ Calcification of the intimal layer is associated with arterial obstruction and atherosclerotic plaque rupture, while calcification of the medial layer is associated with vessel stiffening leading to heart failure.^106^ In addition, vascular calcification has been associated with hypertension, osteoporosis, rheumatoid arthritis, chronic kidney disease, diabetes type II, and aortic aneurysm formation.^106–108^ Interestingly, overexpression of the Twist family BHLH transcription factor 1 (*TWIST1*), a coronary artery disease risk gene, in rat aortic vSMCs increases cell proliferation and decreases calcification, whereas *TWIST1* knockdown has the opposite effect.^109^ Of note, *TWIST1* was also found to be differentially expressed in several vSMC clusters in ascending aorta aneurysm tissue analyzed by scRNA-seq.^75^

The osteogenic-like vSMC phenotype is characterized by a loss of contractile markers (*SM22α* and *ACTA2*) and an increase in calcification markers, such as osteogenic transcription factors MSX2 (Msh Homeobox 2), Cbfa1 (core-binding factor α-1, also known as RUNX2), and Sp7/Osterix, as well as the chondrogenic transcription factor SOX9.^4,106,107,110^ Other markers such as osteopontin, osteocalcin, alkaline phosphatase, collagen II, and collagen X were reported as markers of this osteogenic-like vSMC phenotype as well.^106,111^ The expression of *RUNX2* and *SOX9* is a main determinant of the osteogenic-like vSMC phenotype, since these 2 transcription factors drive the osteoblast or chondrocyte phenotype, respectively.^106,112^

Deficiency of RUNX2 in vSMCs prevents differentiation into the osteogenic-like vSMC phenotype, shown both in vitro and in vivo.^107,113,114^ In vitro, RUNX2, Osterix, and alkaline phosphatase expression is required to drive the calcification process. This process also involves the PKB (protein kinase B) or AKT and c-JNK (Jun N-terminal kinase) signaling pathways. Human vSMCs exposed to a calcifying medium show decreased AKT phosphorylation and a transient increase in JNK activity. This is in line with the observation that elevated levels of AKT and JNK protect from vascular calcification.^115^ Another pathway involves Wnt signaling, which promotes vSMC differentiation to osteogenic-like cells primarily through RUNX2. Activation of the Wnt cascade in osteogenic-like vSMCs increases BMP2 (bone morphogenetic protein 2) signaling,^116^ which plays a significant role in vascular calcification. BMPs are part of the TGF-β superfamily, which signal via the SMAD transcription factors -1 and -5, regulating *RUNX2*.^116^ Interestingly, in disease models associated with vascular calcification, KLF4 was demonstrated to decrease expression of vSMC differentiation makers and induced osteogenic genes.^117^ KLF4 was also demonstrated to regulate RUNX2 transcription, with knockdown of KLF4 inhibiting upregulation of RUNX2 and vSMC calcification.^118^ In addition, KLF4 enhanced chondrocyte differentiation as characterized by upregulation of SOX9 and downregulation of the chondrocyte dedifferentiation marker Col1α1.^119^ In the context of scRNA-seq, loss of KLF4 in vSMCs coincided with reduction of an osteogenic phenotype.^33^ However, the exact link between KLF4 and osteogenic-like cells is not fully clear and requires further research.

An in vivo study recapitulated vSMC differentiation into an osteogenic-like phenotype, by demonstrating that MGP (matrix gla protein)-deficient mice develop severe calcification of vSMCs in arterial blood vessels.^105^ Interestingly, MGP-deficient mice also lacking HDAC9 (histone deacetylase 9) had a 40% reduction in aortic calcification with improved survival.^120^ Thus the presence of HDAC9 causes progression toward the osteogenic-like vSMC phenotype induced by the absence of MGP.

Another pathway involved in the suppression of calcification is based on aryl hydrocarbon receptor (AHR)-mediated gene expression. This transcription factor is involved in stem cell maintenance and cellular differentiation and is a downstream target of TCF21.^99^ TCF21 promotes vSMC phenotype switching to fibroblast-like cells and knockdown of AHR resulted in switching of fibroblast-like vSMCs to the osteogenic-like vSMCs. This is in line with the ability of AHR to suppress *SOX9* and *RUNX2* expression.^99^ Interestingly, TCF21 knockdown prevented both the fibroblast-like and osteogenic-like vSMC differentiation, indicating that the fibroblast-like phenotype may first be required before transition towards the osteoblast-like phenotype can occur.^99^ Taken together, TCF21 can be considered a driver and AHR an inhibitor of these vSMC phenotypes.

## Adipocyte-Like vSMCs

An adipocyte-like vSMC phenotype has only been described in a single scRNA-seq study (Table).^31^ Using an in vivo fate mapping approach, with either a constitutive or an inducible Myh11-driven Cre mouse model, it has been demonstrated that vSMCs are able to adapt toward this adipocyte-like phenotype.^121^ These adipocyte-like vSMC were classified as beige adipocytes, which are also known as inducible brown adipocytes. Brown/beige adipocytes regulate thermogenesis by producing heat when burning fatty acids in the presence of an UCP-1 (uncoupling protein-1), while white adipocytes store triglycerides to save energy.^122,123^ Sustained thermogenic activation leads to the browning of white adipose tissue, in which a population of adipocytes differentiate into beige adipocytes.^123^ Given that only 1 scRNA-seq study reports adipocyte-like vSMCs, this may indicate that a higher barrier exists for differentiation towards an adipocyte-like vSMC type^124^ or that cues to induce this phenotype are not widespread in atherosclerotic and aneurysm tissue.

Adipocyte-like vSMCs express the crucial beige adipocyte marker *UCP1*, as mentioned earlier, as well as PPARγ (peroxisome proliferator-activated receptor gamma) coactivator 1 alpha (*PPARGC1A*), transmembrane protein 26 *(TMEM26*), PR-domain containing 16 (*PRDM16*),^121^ and the temperature-sensitive ion channel transient receptor potential cation channel subfamily V member 1 (*TRPV1*).^125^ Upon 7-day cold exposure, these cells are able to mature further to brown adipocyte-like vSMC expressing *UCP1*, adiponectin (*ADIPOQ*), cell death inducing DFFA like effector A (*CIDEA*), and iodothyronine deiodinase 2 (*DIO2*).^125^ Furthermore, KLF4 is suggested to regulate early adipogenesis to induce C/EBPβ (CCAAT-enhancer-binding protein beta). C/EBPβ stimulates expression of PPARγ, which is required for adipocyte differentiation.^126^ However, if this pathway is also activated in vSMC by KLF4 has yet to be determined.

Conversion of contractile vSMCs to adipocyte-like vSMCs within the vessel wall is presumably not without consequences, but as this phenotype is least studied, this will require more research to understand its relevance.

## Expanding the View on vSMC Phenotypes

**Figure 1.**
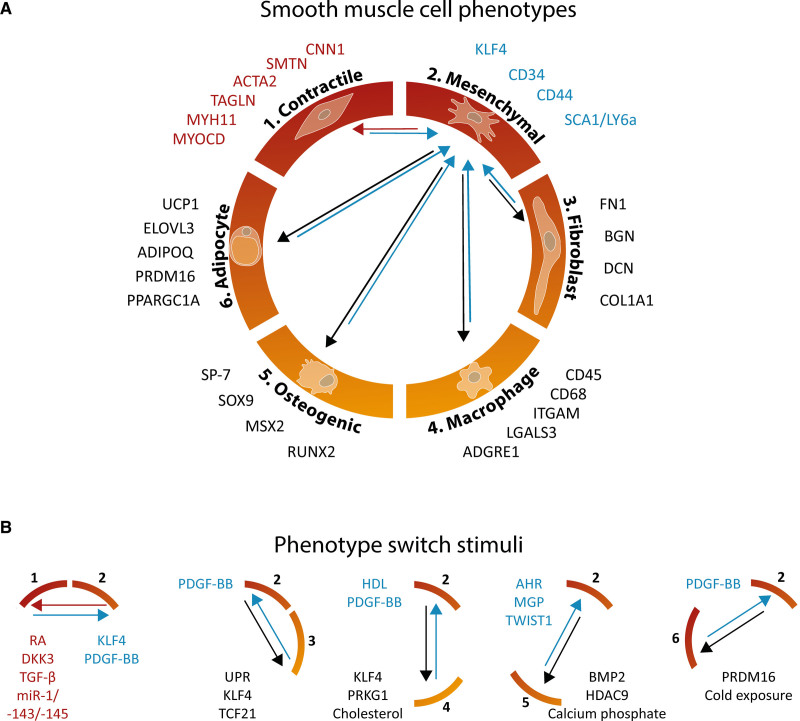
**Schematic overview of vascular smooth muscle cell (vSMC) phenotypes.** **A**, Overview of vSMC phenotypes and associated gene expression markers. The contractile phenotype is associated with the expression of contractile genes. Mesenchymal-like vSMCs lose expression of these contractile markers and adopt expression of a number of specific markers including the key player of phenotype switching: KLF4 (Krüppel-like factor 4). From this vSMC phenotype, differentiation to fibroblast-, macrophage-, osteogenic-, or adipocyte-like phenotypes seems to occur. Genes typical for the contractile phenotype are *CNN1*, *SMTN*, *ACTA2*, *TAGLN*, *MYH11*, and *MYOCD*,^5,7,141,142^ for the mesenchymal phenotype *KLF4*, *CD34*, *CD44, and SCA1/LY6a*,^36,37,143,144^ for the fibroblast phenotype *FN1, BGN*, *DCN*, and *COL1A1*,^7^ for the macrophage phenotype *CD45, CD68, ITGAM, LGALS3*, *ITGAM*, and *ADGRE1*,^7,93,145^ for the osteogenic phenotype *SP-7, SOX9, MSX2*, and *RUNX2*,^106,107,120^ and for the adipocyte phenotype *UCP1*, *ELOVL3*, *ADIPOQ*, *PRDM16*, and *PPARGC1A*.^121^ **B**, Activating and inhibitory stimuli of phenotype switching in vSMCs. Stimuli that mediate specific phenotype switching are indicated. Blue arrows and names indicate a transition to the mesenchymal-like vSMC, red arrows and names indicate a transition to the contractile-like vSMC, and black arrow and names indicate a transition to the other phenotypes in **A** and **B**. AHR indicates aryl hydrocarbon receptor; BMP2, bone morphogenetic protein 2; DKK3, dickkopf 3; HDAC9, histone deacetylase 9; HDL, high-density lipoprotein; MGP, matrix gla protein; PDGF-BB, platelet-derived growth factor BB; RA, retinoic acid; and TGF-β, transforming growth factor beta.

Recent scRNA-seq analyses of human and mouse vascular tissues disclose a previously not well-defined diversity of arterial vSMC phenotypes. We reasoned that the classification of vSMC phenotypes should be expanded beyond the traditional exclusive division of contractile and synthetic vSMCs. In this review, we summarized the current evidence for the presence of 6 distinct vSMC phenotypes in (diseased) vascular tissue: contractile, mesenchymal-like, fibroblast-like, macrophage-like, osteogenic-like, and adipocyte-like vSMCs (Figure 1A). Combining the scRNA-seq data on vSMC subtypes with knowledge on vSMC function, one may speculate that there is an initial transition of the contractile vSMC dedifferentiating into mesenchymal-like vSMCs, followed by differentiation towards the other 4 vSMC phenotypes. A remarkable characteristic of vSMCs is their plasticity, as none of the phenotypes seem to be a final state of cellular differentiation. Rather, vSMCs can go back and forth between different phenotypes, triggered by specific stimuli (Figure 1B). The exact phenotypic composition of vSMCs in the vessel wall may determine vascular pathology in various cardiovascular diseases, and at present the consequence of the relative prevalence of these 6 shades of vSMC is not fully understood. Moreover, presumably there are even more vSMC flavors as indicated by 2 distinct contractile, mesenchymal- and osteogenic-like vSMC populations in different studies,^7,31,33,41,75^ and 8 undefined vSMC populations in the study from Hartman et al.^71^ (Table).

## KLF4 and Its Crucial Role in vSMC Phenotype Switching

The transcription factors KLF4, retinoic acid receptor, TCF21, AHR, and erythroblast transformation-specific related gene are all to some extent involved in vSMC phenotype switching in cardiovascular diseases, with a key role for KLF4. Therefore, we summarize the current knowledge on modulation of KLF4 to interfere with vSMC phenotype switching. KLF4 is known to regulate gene expression via different mechanisms, not merely as DNA-binding transcription factor,^128^ which may in part explain its involvement in the different vSMC phenotypes.

**Figure 2.**
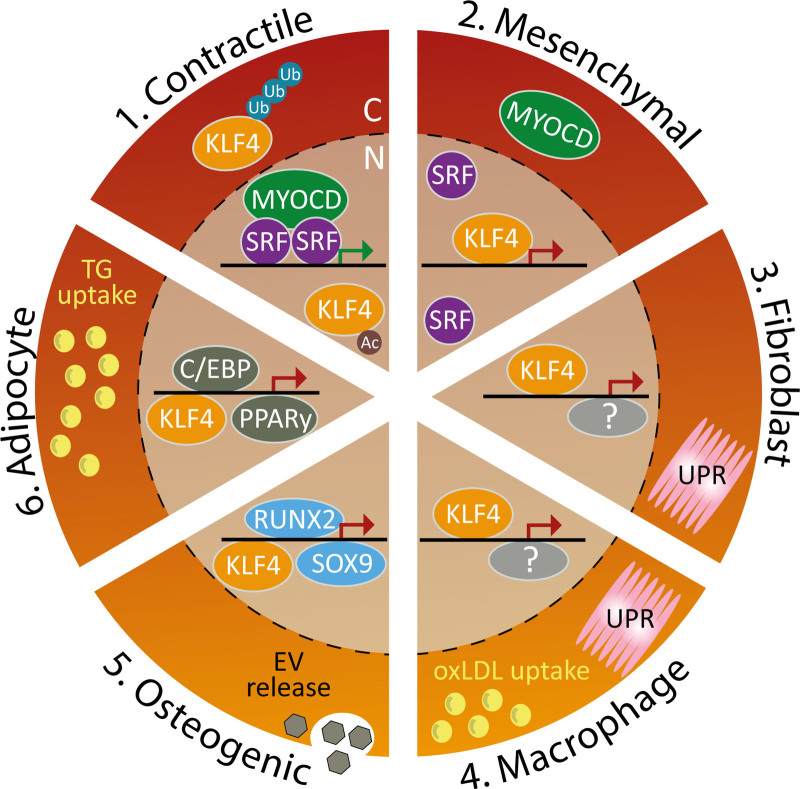
**KLF4 (Krüppel-like factor 4) facilitates phenotypic switching of vascular smooth muscle cells (vSMCs).** Schematic overview of the role of KLF4 in vSMC phenotype switching. In contractile vSMCs (1), the transcription factor complex MYOCD (myocardin)-SRF (serum response factor) induces the expression of contractile genes.^23^ The transcriptional activity of KLF4 is balanced by protein ubiquitination and subsequent degradation. Acetylated KLF4, induced by all-trans retinoic acid stimulation, alleviates the KLF4-induced repression of contractile gene expression.^50,68^ In the mesenchymal vSMC phenotype (2), KLF4 expression is increased and stabilized to repress the expression of contractile genes^50,55,66^ by reduction of formation of the MYOCD-SRF complex.^54^ In the fibroblast-like vSMC (3), the unfolded protein response (UPR) induces KLF4 expression, which affects the vSMC phenotypic switch by an unknown mechanism.^40,99^ The switching toward a macrophage-like vSMC (4) also requires UPR and/or oxidized LDL (oxLDL) uptake to increase KLF4, which by an unknown mechanism induced the switch toward foam cell formation.^40^ The transition to an osteogenic-like vSMC (5) is facilitated by a KLF4-mediated activation of RUNX2 (runt-related transcription factor 2) or SOX9-induced transcriptional programmes, which allows calcification by the release of extracellular vesicles (EVs).^4,106,107,110,127^ The phenotype switch to an adipocyte-like vSMC (6) could hypothetically require activation of KLF4, leading to a KLF4-mediated increase in C/EBP and subsequent PPARy (peroxisome proliferator-activated receptor gamma) transcription factor activation to induce adipogenesis and uptake of triglycerides (TGs).^126^ Green arrow indicates expression of contractile proteins, and red arrow indicates expression of phenotype specific genes. Ac indicates acetylation; C, cytoplasm; N, nucleus; and Ub, ubiquitination.

The sections above substantiate the importance of KLF4 in relation to vSMC phenotype switching. The distinct role of KLF4 per vSMC phenotype with respect to gene regulation and transcription factor cooperation is context-dependent and affected by additional stimuli, as summarized in Figure 2. In the contractile vSMC phenotype, the activated transcription factor complex MYOCD-SRF induces the expression of contractile proteins.^23^ In this situation, KLF4 expression is balanced by protein ubiquitination and subsequent degradation. Posttranslational modification through acetylation of KLF4 changes its binding to preferential DNA sites to alleviate the repression of contractile gene expression. In the mesenchymal-like vSMC phenotype, KLF4 expression is upregulated, which reduces MYOCD-SRF complex formation and thereby inhibits expression of contractile proteins.^50,54,55,66^ KLF4 then increases mesenchymal marker expression: SCA1, CD34, and CD44. When KLF4 signaling is enhanced in the fibroblast-like vSMC phenotype, this is linked to the UPR and its effector factor ATF4.^40^ KLF4 has also been associated with macrophage foam cell formation by enhancing uptake of cholesterol-rich lipoproteins. Switching of vSMCs toward a macrophage-like phenotype may be induced by KLF4 through enhanced UPR or lipid uptake, which can lead to foam cell formation.^40,94^ The transition toward an osteogenic-like phenotype requires the induction of RUNX2 or SOX9 to induce vascular calcification by releasing extracellular vesicles.^4,106,107,110,127^ Activation of KLF4 may be required for switching toward an adipocyte-like phenotype, which could lead to adipogenesis through C/EBPβ and PPARγ signaling.^126^

In atherosclerosis, Alencar et al^33^ identified a reduction in lesion size and increased plaque stability in mice with a vSMC-specific conditional deficiency of KLF4. These mice show an increase in the number of contractile vSMCs, and a substantial reduction of macrophage-like and osteogenic-like vSMCs. Moreover, Chen et al^31^ observed a decrease in overall atherosclerotic burden and a reduction in the development of aortic aneurysms (regardless of cholesterol levels), in vSMC-specific KLF4 knockout mice. A KLF4 signature was also identified in aneurysmal aortic tissue of fibrillin-1 (Fbn1)^C1041G/+^ Marfan syndrome mice.^5^ Furthermore, using an in vitro induced pluripotent stem cell–derived vSMC model with distinct FBN1 mutations from Marfan syndrome patients, knockdown of KLF4 restored Fbn1 fiber deposition, vSMC proliferation defects, and contractile function.^129^ In contrast, a marked reduction in KLF4 expression was observed in the aorta of very young Fbn1 hypomorphic (mgR/mgR model) Marfan mice, which was concomitant with an increase in contractile vSMCs.^130^ Together, these data may indicate that the contractile population of vSMCs is unable to undergo phenotype switching during early stages of aneurysm formation.^5,130^ However, vSMC-specific KLF4-deficient Marfan mice are yet to be generated to precisely determine the effect of KLF4 in aortic aneurysm formation.

These findings emphasize KLF4 as an interesting potential drug target to diminish vascular disease. As there are no highly selective KLF4 antagonists, KLF4 expression can be targeted by indirect inhibitors, of which several are in preclinical development. One of these inhibitors is designed to interfere with the methylation of KLF4 by PRMT5 (protein arginine methyltransferase 5).^131^ Since KLF4 has a short half-life, governed by VHL-VBC ubiquitin-protein ligase, methylation of amino acids R374, R376, and R377 prolong protein stability. The small molecule antagonist WX2–43 was designed to interfere with the PRMT5-KLF4 interaction, preventing the methylation, thus promoting degradation. Another indirect inhibitor of KLF4 targets its interaction with PLK1 (polo-like kinase-1). This kinase phosphorylates KLF4 at Ser234, promoting protein stability, previously linked to hyperplastic intima of injured vessels.^132^ The experimental drug BI6727, originally developed as an anticancer drug, is a highly selective, potent PLK1 inhibitor. Exposure to BI6727 results in reduced expression of KLF4 and increased protein turnover due to reduced stability.^133^ Given that the translation of miRNAs into clinical medicine will become more common in the future, it is of importance that several miRNAs have been reported to interact with KLF4 mRNA, and may serve as blueprint for therapeutic oligonucleotides.^134^ The miRNAs miR-1, miR-25, miR-29, miR-143, miR-145, and miR-375 induce RNAi-mediated silencing of KLF4 protein expression.^92,135–138^ Further research will establish the potential of targeting KLF4 in the context of the different cardiovascular diseases, where KLF4 may be beneficial in one, but detrimental in another vascular disease type. In addition, potential side effects of repressed KLF4 should not be overlooked, as KLF4 inhibition has been shown to delay wound healing and elevate insulin resistance.^139,140^

## Discussion and Perspectives

The scRNA-seq technique, together with lineage tracing, opens up a whole new world of possibilities to study vSMCs in the normal and diseased vessel wall. Until now, the scRNA-seq has been performed on (diseased) tissues collected at a single point in time, especially in human end-stage disease. To understand the dynamics of vSMC phenotype plasticity between the 6 vSMC phenotypes, phenotype switching over time needs to be explored in detail. To date, only Pan et al^8^ performed scRNA-seq and lineage tracing in mice over various time points and suggested that this method provided both sensitive and specificity for tracking vSMC behaviors during atherosclerosis.

In addition, we argued that the data point in the direction of the mesenchymal-like cells giving rise to the other vSMC phenotypes; however, further experimental validation is needed to fully support this hypothesis. Furthermore, it remains challenging to recognize the vSMC origin of a cell population, especially in nonlineage traced human tissue samples. However, even conventional lineage tracing studies have their limitations; they can only mark the fate of the contractile vSMC but not the fate of once contractile vSMCs that underwent phenotype switching with the loss of their contractile markers.

Limitations and potential biases should also be considered when analyzing and interpreting different scRNA-seq datasets. As often, the data rely on a single-cell suspension representative of all cell populations present within tissue samples exposed to tissue disruption. scRNA-seq also lacks spatial information about the distribution of the different subpopulations within tissues, and different processes can cause variations in transcript levels that might not reflect differences in protein levels or cellular functions. Moreover, the lack of depth of scRNA-seq also makes it difficult to investigate low-expression genes, which are often transcription factors.^10^

Although the transcriptomic signature in defining vSMC states and transitions offers a wealth of information, it is yet to be determined what this means on a protein level and whether these vSMC-derived cells have similar functionality to their classical counterparts (eg, macrophage-like vSMC compared with a bone marrow-derived macrophage). The vSMC-derived cells seem to be heavily influenced by their environment to trigger the transition and may not always provide positive consequences.

As for the function of the different vSMCs phenotypes, their diverging roles in the broad range of CVDs remain to be discovered. It will eventually pinpoint which type is beneficial or harmful in specific vascular disorders. This knowledge is crucial for the development of innovative disease intervention strategies.

## Article Information

### Acknowledgment

We thank Dr Dave Speijer for critical reading of the article.

### Sources of Funding

This study was supported by Amsterdam Cardiovascular Sciences PhD grant 2019 and Zeldzame Ziekten Fonds via AMC foundation (C. Yap, D. Micha, V. de Waard) and by Health Holland TKI-Public Private Partnership grant 22532 (A. Mieremet, V. de Waard).

### Disclosures

None.
